# Association between Germline Single-Nucleotide Variants in ADME Genes and Major Molecular Response to Imatinib in Chronic Myeloid Leukemia Patients

**DOI:** 10.3390/jcm11206217

**Published:** 2022-10-21

**Authors:** Natalia Estrada, Lurdes Zamora, Francisca Ferrer-Marín, Laura Palomo, Olga García, Patricia Vélez, Iris De la Fuente, Miguel Sagüés, Marta Cabezón, Montserrat Cortés, Rolando Omar Vallansot, María Alicia Senín-Magán, Concepción Boqué, Blanca Xicoy

**Affiliations:** 1 Myeloid Neoplasms Group, Josep Carreras Leukaemia Research Institute, ICO-Hospital Germans Trias i Pujol, Universitat Autònoma de Barcelona, 08916 Badalona, Spain; 2 Hospital General Universitario Morales Meseguer, CIBERER (CB15/00055), IMIB-Pascual Parrilla, UCAM, 30008 Murcia, Spain; 3 MDS Group, Josep Carreras Leukaemia Research Institute, ICO-Hospital Germans Trias i Pujol, Universitat Autònoma de Barcelona, 08916 Badalona, Spain; 4 Experimental Hematology, Vall d’Hebron Institute of Oncology (VHIO), 08035 Barcelona, Spain; 5 ICO-Hospital Duran y Reynals, 08908 Barcelona, Spain; 6 ICO-Hospital Josep Trueta, 17007 Girona, Spain; 7 Hospital de Granollers, 08402 Granollers, Spain; 8 ICO-Tarragona, Hospital Joan XXIII, 43005 Tarragona, Spain

**Keywords:** chronic myeloid leukemia, imatinib, major molecular response, single-nucleotide polymorphisms

## Abstract

Imatinib is the most common first-line tyrosine kinase inhibitor (TKI) used to treat chronic-phase chronic myeloid leukemia (CP-CML). However, only a proportion of patients achieve major molecular response (MMR), so there is a need to find biological factors that aid the selection of the optimal therapeutic strategy (imatinib vs. more potent second-generation TKIs). The aim of this retrospective study was to understand the contribution of germline single-nucleotide variants (gSNVs) in the achievement of MMR with imatinib. In particular, a discovery cohort including 45 CP-CML patients was analyzed through the DMET array, which interrogates 1936 variants in 231 genes related to the absorption, distribution, metabolism and excretion (ADME) process. Variants statistically significant in the discovery cohort were then tested in an extended and independent cohort of 137 CP-CML patients. Finally, a total of 7 gSNVs (*ABCG1*-rs492338, *ABCB11*-rs496550, *ABCB11*-rs497692, *CYP2D6*-rs1135840, *CYP11B1*-rs7003319, *MAT1A*-rs4934027 and *SLC22A1*-rs628031) and one haplotype in the *ABCB11* gene were significantly associated with the achievement of MMR with first-line imatinibtreatment. In conclusion, we identified a genetic signature of response to imatinib in CP-CML patients that could be useful in selecting those patients that may benefit from starting imatinib as first-line therapy, therefore avoiding the toxicity related to second-generation TKIs.

## 1. Introduction

Tyrosine kinase inhibitors (TKIs) have radically changed the outcome of chronic myeloid leukemia (CML) patients in the last 20 years. Imatinib, the first TKI developed, is capable of inducing complete hematologic and cytogenetic response in the majority of chronic-phase CML (CP-CML) patients, and the goal of CML therapy has moved to the achievement of a major molecular response (MMR; defined as BCR::ABL1/ABL1 ratio ≤0.1% on the international scale (IS)) and possibly the achievement of a deep molecular response (DMR; defined as BCR::ABL1/ABL1^IS^ ratio ≤0.01%), which might represent an “operational cure” as well as a prerequisite for treatment discontinuation [1,2]. Despite the excellent efficacy and improved clinical response obtained with imatinib, development of resistance in a significant proportion of CML patients on imatinib therapy has emerged as a challenging problem in clinical practice. The spectrum of therapeutic options for CML patients has been enriched by second-generation TKIs, such as nilotinib, dasatinib and bosutinib, all of them approved for first-line treatment and being more potent and/or selective than imatinib in BCR::ABL1 inhibition. However, these second-generation TKIs often present high toxicity compared to imatinib, so clinicians must carefully select the best frontline choice of drug for CML treatment in order to minimize adverse events.

One of the main research focuses on CML has been the identification of biological predictors related to TKI response, allowing treatment optimization. Differences observed in TKI efficacy are mainly due to its inhibitory potency of the BCR::ABL1 oncoprotein as well as the acquisition of point mutations in *ABL1* kinase domain that leads to an inefficient binding of the TKI to its target, the latter accounting for 30–40% of imatinib-resistant cases [3,4]. However, as with many other drugs, TKIs present an elevated interpatient variability that results in different drug bioavailability, which in turn influences plasma and intracellular concentrations and finally affects the therapeutic response. Germline single-nucleotide variants (gSNVs) in genes involved in drug pharmacokinetics, i.e., drug metabolism and transport (known as the ADME process: absorption, distribution, metabolism and excretion) are likely to be the most important sources of individual variability in drug efficacy [5].

Imatinib, along with other TKIs, is a substrate for solute carrier transporters (SLCs), such as human organic cation transporter 1 (hOCT1, encoded by the *SLC22A1* gene), organic cation transporter 2 (OCTN2, encoded by *SLC22A5*), organic anion-transporting polypeptide 1A2 (OATP1A2, encoded by *SLCO1A2*) [6] as well as organic anion-transporting polypeptide 1B3 (OATP1B3, encoded by *SLCO1B3*), all of them participating in the active uptake of imatinib into cells [7]. On the other hand, TKI efflux is mediated by the ABC transporters, in particular ABCB1 (also known as multidrug resistance protein 1, MDR1) and, to a lesser extent, ABCG2 (also known as breast cancer resistance protein, BCRP) [8]. Moreover, metabolization of imatinib, and almost all TKIs, occurs in the liver via the cytochrome-P450, mostly CYP3A4 and 3A5 isoforms [9]. Changes in the functionality of these proteins have been linked to gSNVs in their coding genes that could play an important role in imatinib disposition and therapeutic response.

Previous studies have analyzed the influence of gSNVs in ADME genes and the response to TKI treatment, mainly imatinib. However, most of them show limitations in patient selection criteria, such as including heterogeneous populations of patients in different CML phases or treated with different TKIs at first-line as well as different treatment doses [10,11,12,13,14,15]. In addition, few gSNVs, identified through a candidate gene approach, have been proposed as involved in imatinib response [15,16,17,18]. Besides the metabolizers and transporters mentioned above, other enzymes may be implicated in the imatinib ADME processes. To explore this hypothesis and due to the relevance of determining the factors that influence variability in response to CML treatment, the aim of this study was to evaluate genetic variants that may be involved in the achievement of MMR with first-line imatinib treatment by using a more complete multi-gene approach through the drug-metabolizing enzyme and transporter (DMET) genetic platform (Affymetrix). Indeed, this platform has been used in different cancer populations and other diseases [19,20,21] and is capable of simultaneously investigating 1936 genetic variants in 231 ADME genes [22]. With this premise, we performed the present retrospective study by using the DMET genetic platform in the context of a multicenter “real-life” discovery set of 45 CP-CML patients treated with first-line imatinib. Afterwards, those SNVs significantly associated with the achievement of MMR in the discovery set were tested in an independent and multicenter “real-life” cohort of 137 CP-CML patients also receiving imatinib as first-line treatment.

## 2. Materials and Methods

### 2.1. Study Population

A total of 182 CP-CML patients, collected between 2005 and 2016, were retrospectively included in the study; all patients were of Caucasian ethnicity and treated with imatinib 400 mg daily as first-line treatment. In particular, 45 out of 182 patients were used as the discovery set and 137 as an extended cohort. The study was approved by the local Ethical Committee of the Germans Trias i Pujol Hospital, Badalona, Barcelona. Genomic analysis was performed after obtaining written informed consent for study participation in accordance with national legislation and the Helsinki Declaration. All clinical information was collected from the patient’s medical records. Table 1 summarizes selected demographic and clinical characteristics of our study population. Both the discovery set and the extended cohort were comparable. Patients were retrospectively selected according to the following criteria: (1) a minimum of 1 year follow-up, (2) absence of toxicity or intolerance to imatinib that required a change of TKI treatment or a dose reduction and (3) in case of imatinib failure, absence of previously identified mutations in the *ABL1* gene or additional cytogenetic abnormalities to avoid any statistical confounding bias.

### 2.2. Response Definition and Patient Classification

Bone marrow morphology and cytogenetic studies were performed on bone marrow samples to confirm the diagnosis and to categorize the CML phase. BCR::ABL1 transcript levels were measured in peripheral blood by real-time qPCR, as previously described [23]. *ABL1* was used as the endogenous control gene, and the results were reported as %BCR::ABL1/ABL1 on the IS, and molecular response (MR) monitoring was performed at diagnosis (baseline) and every 3 to 6 months of imatinib treatment thereafter.

In the present retrospective case-control study, we applied the following classification criteria: for the discovery set, patients were retrospectively classified as “non-responders” if they had failed to achieve MMR at 12 months of imatinib initiation, so only patients with an optimal MR according to European LeukemiaNet 2013 (ELN2013) recommendations [24] were included as “responders”. On the other hand, patients in the extended cohort were classified as “non-responders” if they had failed to achieve a BCR::ABL1/ABL1^IS^ ratio <10% at 6 months or a BCR::ABL1/ABL1^IS^ ratio <1% at 12 months with imatinib as first-line treatment, according to ELN2013 recommendations for TKI treatment failure. In the extended cohort, patients considered as “responders” were those with an optimal response or those with “warning” criteria—as described by ELN2013—that did not change imatinib dose or switch to another TKI. All the patients in this group eventually achieved MMR with first-line imatinib treatment. The reason for applying different classification criteria in our two cohorts was that in the discovery set, we aimed to select only the gSNVs that allowed us to differentiate between very good responders (achievement of MMR at 12 months) and poor responders (ELN2013 “warning” and/or failure criteria). However, in the extended cohort, only patients that changed from imatinib to a different TKI due to ELN2013 failure criterion (and not “warning” criterion) were considered as “non-responders”, thus better resembling the clinical practice.

### 2.3. Genotype Analysis in the Discovery Set and in the Extended Cohort

DNA was isolated from whole blood using QIAcube^®^ (Qiagen, Hilden, Germany) with the QIAamp DNA Blood Mini Kit (Qiagen). DNA concentration and quality status were assessed with the QIAxpert System (Qiagen). The Affymetrix DMET Plus Premier Pack (Affymetrix, Santa Clara, CA, USA) was used to genotype DNA from the 45 CP-CML patients included in the discovery set [22], which allows the analysis of up to 1936 variants in 231 adsorption, distribution, metabolism and excretion (ADME) genes. Selected gSNVs significantly associated with response to imatinib (*p* < 0.05) in the discovery set were then tested in an independent cohort of 137 CP-CML patients using the 96.96 Dynamic Array^TM^ integrated fluidic circuit (IFC) with SNP Type Assay chemistry on the Biomark HD system (Fluidigm, South San Francisco, CA, USA), with previous pre-amplification of all the DNA samples and following the manufacturer’s instructions.

For both genotyping platforms, call rates less than 95% were excluded from further analysis. The accuracy of genotyping was confirmed by performing triplicates of all the samples, all of them being 100% concordant.

### 2.4. Statistical and Bioinformatics Analysis

The study group characteristics were described as frequency and percentage for categorical variables and median and range for quantitative variables. Comparisons of continuous variables between groups were made using the median test, while categorical variables were compared using the χ^2^ test or Fisher exact test, if necessary.

**Figure 1:**
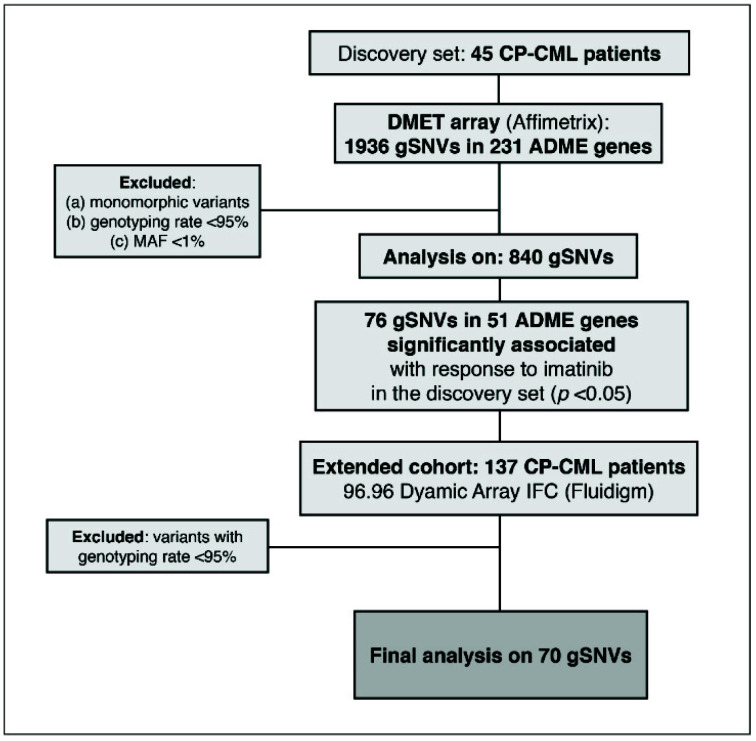
Flowchart describing genetic marker selection. ADME: adsorption, distribution, metabolism and excretion; MAF: minor allele frequency. gSNV: germline single-nucleotide variant.

Regarding genotyping data, and as shown in the flowchart in Figure 1, we first performed a quality control check of the initial 1936 variants and excluded those that were monomorphic as well as those with a minor allele frequency (MAF) <1%. Then, we tested the Hardy–Weinberg equilibrium (HWE) to further eliminate gSNVs deviating from the equilibrium (*p* ≤ 0.05). After the quality control check, gSNVs were correlated with the achievement of MMR with first-line imatinib treatment using the *SNPassoc* package from R software [25] which provides the *p*-value for the likelihood ratio test of association (or Fisher’s exact test if some cell is empty), odds ratios with 95% confidence intervals (CIs) and the Akaike information criterion, assuming different genetic models. Bonferroni correction for multiple comparisons was implemented in all the analyses.

Only in the analysis of the extended cohort and with the aim of statistically validating our previous results, we performed a cross-validation analysis and a permutation test. Cross-validation uses different portions of the data to test and train a model on different iterations. In the present study, we randomly divided our sample into 5 equal groups and performed the same association analysis up to 5 times, always leaving out one of the groups. The permutation test was carried out to check the significance of the univariate associations of the selected gSNVs with the achievement of MMR at any time. In this type of non-parametric test, genotypes are permuted relative to affection status (in this case, MMR achievement as case or control status) to produce many random datasets, and the process of training and recording of outputs is repeated. We permuted the case/control status for all the individuals up to 10,000 times and estimated the association *p*-values for all gSNVs with the logistic regression model.

In addition, those gSNVs statistically significant in the extended cohort for the abovementioned analyses were studied by cumulative incidences considering MMR as a time-dependent variable. In this case, competing risk analysis was applied by using the Fine and Grey model (for which achievement of the MMR was the event of interest, and competing risks were any event that led to permanent cessation of first-line imatinib).

Finally, haplotype blocks between gSNVs were inferred using the Haploview software package [26], and their association with imatinib response was evaluated using the Haplo.stats package in R v4.0.1 software.

Two-sided *p*-values < 0.05 were considered statistically significant. The statistical packages SPSS version 24.0 (SPSS Inc., Chicago, IL, USA) and R v4.0.1 software (R Foundation for Statistical Computing, Vienna, Austria) were used for all the analyses and the creation of graphics.

## 3. Results

### 3.1. gSNVs Associated with MMR Achievement in the Discovery Set

Regarding patients’ classification criteria for the discovery set (*n* = 45), 17 (38%) patients achieved MMR at 12 months of starting imatinib and were considered as “responders” (controls), whereas 28 (62%) did not achieve MMR at 12 months and were considered as “non-responders” (cases).

From the initial 1936 variants tested in the DMET Affymetrix array, 1096 of them were excluded due to homozygosity for the wild-type allele, MAF < 1% and/or a genotyping rate <95%. A total of 840 variants fulfilled the criteria and were tested for association with response to imatinib. The analysis highlighted that 76 gSNVs, in 51 different genes, showed a significant association with the achievement of MMR at 12 months with first-line imatinibtreatment in the discovery set (Figure 1 and Table S1).

### 3.2. gSNVs Associated with MMR Achievement in the Extended Cohort

The extended cohort included a total of 137 CP-CML patients, of whom 37 (27%) were considered as “non-responders” (cases) and 100 (73%) were considered as “responders” (controls). All the patients included in the category of “responders” achieved MMR (most of them before 20 months of imatinib treatment). The 76 gSNVs previously associated with imatinib response in the discovery set were genotyped using the Biomark HD platform. Six SNVs (rs743616, rs11770903, rs2884737, rs17685, rs12960, rs13226149) were excluded due to a genotyping rate <95%. Finally, the association analysis included a total of 70 gSNVs and was performed in 137 CP-CML patients (extended cohort). Seven gSNVs in six different genes showed a significant association with MMR achievement at any time in the extended cohort (Table 2).

The presence of two copies of the minor allele A for rs628031, located in the SLC22A1 influx transporter gene, showed lower rates of MMR achievement compared to patients with G/G and A/G genotypes for this gSNV (*p* = 0.015). Moreover, patients carrying one or two copies of the C allele in rs492338, a gSNV located in another transporter gene, the ABCG1, were more likely to achieve MMR (*p* = 0.021). Two gSNVs present in the transporter coding gene ABCB11, rs496550 and rs497692, also showed an association; in 87% and 75% of patients with A/A and A/G genotypes, respectively, and for both gSNVs, MMR was achieved with first-line imatinib, whereas only 62% of patients harboring the G/G genotype for both gSNVs achieved MMR (*p* = 0.027). These two gSNVs showed practically equal genotype frequencies as well as odds ratios, indicating a possible genetic linkage by being inherited together.

Two gSNVs in different genes of the cytochrome P450 family were significantly associated with imatinib treatment; the presence of two copies of the minor allele G for rs1135840, located in the CYP2D6 gene, was associated with high risk of MMR failure (*p* = 0.037). Of note, from all the statistically significant gSNVs, rs1135840 was the only one that did not fulfill HWE (*p* = 0.019) but was included in the final analysis due to its possible relevance to leukemogenesis [27] (discussed in more detail further on). Patients with genotypes A/C and A/A for rs7003319, located in the CYP11B1 gene, showed lower rates of MMR achievement than patients with the C/C genotype (66 vs. 81%, respectively, *p* = 0.038).

Finally, the gSNV rs4934027 located in the MAT1A gene, showed a significant association with imatinib response, since 88% of patients with genotypes C/T and T/T achieved MMR as opposed to 64% of patients with the C/C genotype (*p* = 0.002).

### 3.3. Validation of the Results Obtained in the Extended Cohort by Cross-Validation Analysis and Permutation Test

Two additional association analyses were performed for the extended cohort to validate the significant impact of gSNVs on MMR achievement at any time with first-line imatinib treatment. Cross-validation analysis was used to guarantee that significant results were independent of the partitions applied to our study sample. The extended cohort was randomly divided into a total of five partitions of the same sample size (27–28 patients per group) and the association analysis was performed up to five times, always retaining one of the partitions out of the analysis. Therefore, the five association analyses included a total of 110–111 patients, of which 29–30 (26–27%) were classified as non-responders (cases).

Table 3 shows the gSNVs significantly associated with MMR achievement obtained in each of the five analyses performed, with results grouped in A, B, C, D or E sets. All the seven significant gSNVs obtained in the first association study with the entire cohort (Section 3.2) were also significant in one or more of the association analyses in the cross-validation test, which reinforces our results. Additionally, ABCB11 rs495714 was significant in one of the groups but did not show significance in the association analysis for the total cohort. MAT1A rs4934027 was the only one statistically significant in all the analysis groups, thus confirming that patients with C/T and T/T genotypes for this gSNV are more likely to achieve MMR with first-line imatinib.

Regarding the permutation test, we observed that all the gSNVs statistically significant in the analysis with the entire cohort (Section 3.2) maintained a significant association with MMR achievement after performing 10,000 iterations of the case-control status. Moreover, two new gSNVs, rs4148304 at the UGT2A1 gene and rs1065852 at the CYP2D6 gene, were significant only in this analysis (*p* = 0.012 and *p* = 0.003, respectively) (Table 4).

### 3.4. Cumulative Incidences of MMR Achievement for the Selected Significant gSNVs Obtained in the Extended Cohort

For all the gSNVs significantly associated with MMR achievement in the different analyses performed for the extended cohort, we additionally studied the cumulative incidence of achieving MMR at any time to assess the impact of the different gSNVs and their genotypes from imatinib initiation and during follow-up.

**Figure 2:**
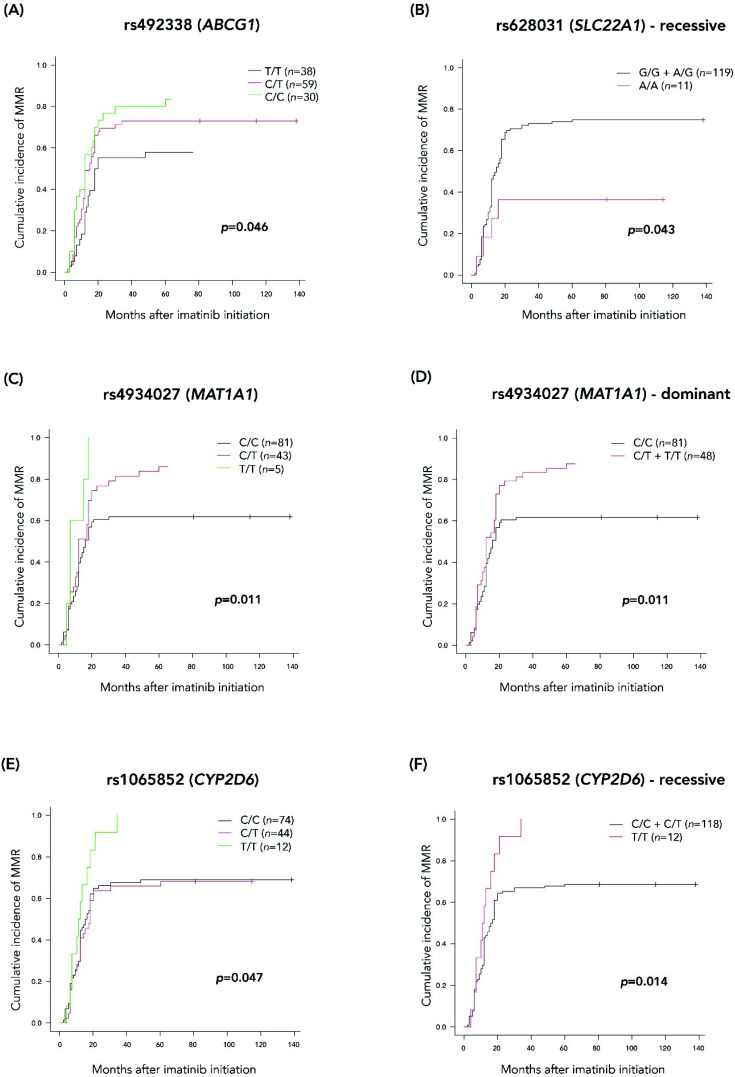
Cumulative incidence (95% CI) of MMR for the gSNVs with *p*-value < 0.05. (**A**) 95% CI of MMR for rs492338 (ABCG1) for each of the three genotypes. (**B**) 95% CI of MMR for rs628031 (SLC22A1) grouped by recessive model. (**C**,**D**) 95% CI of MMR for rs4934027 (MAT1A) for each of the genotypes and grouped by dominant model. (**E**,**F**) 95% of MMR for rs1065852 (CYP2D6) for each of the genotypes and grouped by recessive model.

In this case, six patients were excluded from the analysis due to the lack of the exact date of MMR achievement. Cumulative incidences with 95% CI for each gSNV are shown in Table S2. Only the gSNVs that showed a significant association (*p* < 0.05) with MMR achievement as a time-dependent variable were graphically represented (Figure 2). For those cases in which the significant hereditary model is recessive or dominant, cumulative incidences as well as graphs are represented for both the three genotypes separately and the re-grouped genotypes.

Of note, patients carrying the A/A genotype for rs628031 (SLC22A1) showed a significant decrease in MMR cumulative incidence, as well as patients with at least one copy of the T allele for rs4934027 (MAT1A). In contrast, patients carrying two copies of the minor allele T for rs1065852 (CYP2D6) had a significant advantage in achieving MMR with first-line imatinib treatment.

### 3.5. Haplotype Analysis and Association with MMR Achievement

**Figure 3:**
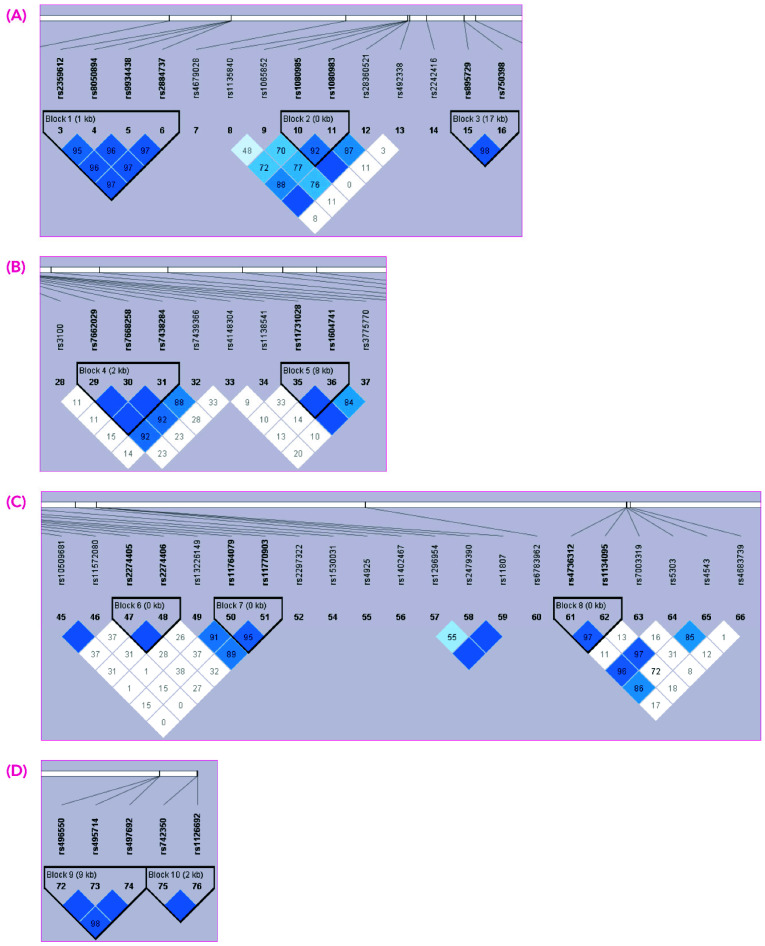
Haploview linkage disequilibrium graph. A total of 10 haplotype blocks were identified (**A**–**D**). Block 9 (**D**) was the only one statistically associated with the achievement of MMR with imatinib.

Finally, we investigated the presence of haplotypes within the 70 selected gSNVs analyzed in the extended cohort. Using Haploview software [26], we found a total of 10 haplotype blocks (Figure 3). We then evaluated the association of these haplotype blocks with the response to imatinib. Logistic regression analysis revealed that only one haplotype was significantly associated with the achievement of MMR: block 9 on chromosome 2 (Figure 3D), formed by rs496550, rs495714, rs497692 gSNVs of the ABCB11 gene. In particular, carriers of the ABCB11-AGA haplotype, which was present in 45% of our extended cohort, had a higher probability of MMR achievement with first-line imatinib treatment, compared with the most frequent ABCB11-GAG haplotype, which was present in 54% of our extended cohort (OR (95% CI): 0.51 (0.28—0.93), *p* = 0.027).

## 4. Discussion

Imatinib is a specific inhibitor of the BCR::ABL1 fusion protein and is an example of successful targeted therapy, still being the treatment of choice for most of de novo CML patients with low-risk disease. Successful identification of genetic markers of imatinib response would provide a risk evaluation tool for stratification of CML patients at diagnosis, paving the way for a personalized therapeutic approach. To this end, a refined stratification of patients in terms of likelihood of achieving MMR would be the necessary starting point, enabling the identification of those patients who are more likely to benefit from imatinib rather than from a more toxic second-generation TKI and helping to evaluate the risk/benefit balance. Polymorphic variants in genes involved in imatinib mesylate pharmacokinetics may explain at least part of the interindividual variability of imatinib response in CML patients [28].

In this study we moved, for the first time in CML, from testing a few gSNVs in a limited number of genes to the application of a pharmacogenetics platform, namely the Affymetrix DMET array, which allows the simultaneous analysis of a wide panel of genetic variants in genes involved in the ADME process. Indeed, the previously published literature on pharmacogenetics in CML used a multiple gene candidate approach although only focused on specific gSNVs and, as a downside, is likely to miss important significant results. Moreover, as opposed to previously published studies that included different treatment approaches, we included a homogeneous cohort of only CP-CML patients treated with imatinib 400 mg/day as first-line therapy [10,11,12,13,14,15,16,17,18]. In particular, our study included two different real-life practice patient cohorts; the first one (*n* = 45) was used as the discovery set, in which the DMET array was applied. Of note, 62% of patients in the discovery set were classified as “non-responders” due to the classification criteria used for this cohort, where only patients with optimal MR were considered as “responders”, thus not corresponding with the percentage described in the literature [29]. A total of 76 gSNVs were significantly associated with MMR achievement with imatinib treatment in this first cohort and were included in the analysis with the extended cohort (*n* = 137), in which 27% of patients were considered as “non-responders”, thus resembling the results from the literature [29].

Overall, after the confirmation step in this extended cohort and deepening the investigation through cross-validation and permutation analyses, we identified seven gSNVs in six genes—*ABCG1* rs492338, *ABCB11* rs496550, *ABCB11* rs497692, *SLC22A1* rs628031, *CYP2D6* rs1135840, *CYP11B1* rs7003319 and *MAT1A* rs4934027—significantly associated with the achievement of MMR with imatinib first-line treatment.

Several studies have reported that genetic variants in influx transporters, such as SLC22A1, as well as efflux transporters, such ABCG2 and ABCB1, may be associated with imatinib levels. Among the *SLC22A1* gene, the most commonly reported gSNVs are rs628031, rs683369 and rs35191146. Takahashi et al. [30] found an association between genotype GG of rs628031 and the achievement of MMR with imatinib treatment in a cohort of 67 Japanese CML patients. These results were in concordance with the findings observed by Koren-Michowitz et al. [16] and Vaidya et al. [18]. In our study, 25 gSNVs present at the *SLC22A1* gene were tested with the DMET array and only rs628031 showed an association with imatinib response, both in the discovery set and the extended cohort. This gSNV represents a non-synonymous variant that promotes the change of methionine for valine at position 408 (M[ATG] > V[GTG]). Our results suggest that carrying two copies of the A allele decreases the probability of MMR achievement with imatinib, which is in line with the abovementioned studies in addition to the extended meta-analysis published by Cargnin S. et al. [31]. Notably, this gSNV has also been associated with decreased response to metformin in patients with type II diabetes mellitus [32].

Furthermore, ATP-binding cassette (ABC) transporters are a family of enzymes involved in the efflux of different drugs. Several studies have reported that genetic variants in these efflux transporters, such as ABCG2 and ABCB1, may be associated with imatinib plasma levels [8,33]. Among all the gSNVs in *ABCG2* and *ABCB1* genes included in the DMET array, none of them showed a significant association with imatinib response in our discovery set. However, different gSNVs in other ABC transporters, such as ABCG1 and ABCB11, did show an association and were included in the analysis of the extended cohort. One gSNV in *ABCG1*, rs492338, showed an association with MMR achievement in all the statistical and bioinformatics analyses performed. This gSNV represents an intronic variant; hence, it does not imply any change in the protein sequence but may have a role in the correct generation of the encoded protein. In our study, patients carrying two copies of the T allele showed lower rates of MMR compared to patients with one or two copies of the C allele (Figure 2A). Since ABCG1 is implicated in the active transport of cholesterol and other lipoproteins [34,35], together with the observed synergistic effect of imatinib and antilipidemic drugs in anti-leukemia activity [36,37], we hypothesize that genetic variations in *ABCG1* play a role in imatinib excretion mediated by possible changes in the lipidic composition of the cellular membrane. Besides *ABCG1*, we found an association with three gSNVs in the *ABCB11* gene and imatinib response; of these, rs496550 and rs497692, which represent an intronic and a synonymous variant, respectively, were significant in all the analyses. Additionally, these two gSNVs together with the rs495714 gSNV belonged to a haplotype that was statistically linked to MMR achievement. Patients harboring the *ABCB11*-AGA genotype (loci rs496550, rs495714 and rs497692, respectively) showed higher probability of MMR achievement with first-line imatinib treatment. As far as we know, none of the pharmacokinetic studies of imatinib has pointed out an involvement of the ABCG1 and ABCB11 proteins in imatinib transport; however, it cannot be excluded that these transporters are directly or indirectly involved in imatinib uptake. Even though we have performed several statistical analyses to avoid random results, we cannot completely exclude the possibility that the associations found in our study occurred by chance, making it essential to replicate the observation in an independent dataset.

Besides drug transporters, proteins related to imatinib metabolization have been associated with therapeutic response. Among them, two members of cytochrome P450, CYP3A4 and CYP3A5, have been widely described as major contributors to this process [9]. Other isoforms such as CYP2C8, CYP2C9 and CYP2D6 have also been related to imatinib metabolism, although to a lesser extent. Different studies have demonstrated the association of specific gSNVs in the genes coding for these proteins and imatinib response rates [11,15,38,39]. In our study, among the gSNVs in CYP450 isoforms tested with the DMET array, 17 showed a significant association with imatinib response in the discovery set and were included in the extended cohort. Finally, only two of these 17 gSNVs were significantly associated with MMR achievement in the extended cohort: one of them was rs7003319 in the *CYP11B1* gene, which encodes a variant present in the regulatory region 3’UTR and had a significant association with MMR in the dominant model, since patients carrying A/C or A/A had lower rates of MMR achievement. As far as we know, neither this gSNV nor the CYP11B1 enzyme have been related to CML, but Ravegnini et al. [40] described an association of rs7003319 and higher survival rates in patients with gastrointestinal stromal tumor treated with imatinib. The other gSNV was rs1135840 from *CYP2D6*, which represents a change of serine for threonine at position 486 (S[AGC] > T[ACC]). However, this gSNV did not fulfill HWE; a possible explanation for this phenomenon is that HWE is a population property, and in many human genetics studies, subjects are ascertained through their disease status (in this case, leukemia), so that affected individuals are more represented in the sample than in the general population. As a result, when a marker is associated with the disease, the corresponding genotypes may no longer be a random sample [41]. In fact, as we did not include any healthy control in our study, we may have selected a specific genotype associated with CML and/or leukemogenesis [27].

Finally, the gSNV rs4934027 in the *MAT1A* gene was significant in all the statistical analyses performed, being the one with the lowest *p*-value in our extended cohort. Neither this gene nor this gSNV have been previously described in CML or in imatinib metabolism, and considering that rs4934027 represents an intronic variant, its role in imatinib resistance should be further investigated.

Due to the retrospective nature of this study, there are some limitations to consider; first, imatinib plasma levels were not tested, and concomitant treatment was not available, which may have affected the imatinib response in both cases. Second, although those patients would eventually benefit from a different TKI as first-line therapy, the influence of such gSNVs with second-generation TKIs should be also studied to be sure that those drugs in first-line are the alternative to imatinib.

In conclusion, to the best of our knowledge, this study represents the first example of DMET array application in CML patients, which allowed us to interrogate multiple gSNVs in a previously selected uniform cohort. Data reported here are promising and, if validated in a larger cohort, would be of great value for selecting those patients who are likely to benefit from starting imatinib as first-line therapy (i.e., MMR achievement), avoiding the toxicity related to second-generation TKIs and promoting patient’s adherence to treatment as well as improving the cost–benefit balance due to the high cost of second-generation TKIs compared to generic imatinib.

## Figures and Tables

**Table 1:** Patients’ characteristics divided by the discovery set and the extended cohort.

Clinical Characteristics		Discovery Set (*n* = 45)	Extended Cohort (*n* = 137)
Median age at diagnosis, y (range)		52 (19–73)	52 (23–86)
Sex, *n* (%)	Female	21 (47)	57 (42)
	Male	24 (53)	80 (58)
Sokal, *n* (%)	Low risk	20/42 (48)	61/127 (48)
	Int ^1^ risk	18/42 (43)	47/127 (37)
	High risk	4/42 (9)	19/127 (15)
ELTS ^2^, *n* (%)	Low risk	26/39 (67)	53/84 (63)
	Int risk	11/39 (28)	23/84 (27)
	High risk	2/39 (5)	8/84 (10)
Imatinib treatment duration, m (range)		52 (6–142)	78 (6–208)
Median follow-up, y (range)		13 (5–18)	13 (1–19)
Exitus, *n* (%)		5 (9)	21 (15)

^1^ Int: intermediate; ^2^ ELTS: EUTOS long-term survival score.

**Table 2:** SNVs significantly associated (*p* < 0.05) with MMR achievement with imatinib first-line treatment in the extended cohort. Genetic model of inheritance and genotype are indicated in each case.

Gene	RefSNP ID	Location	Model ^a^	Genotype	N Cases ^b^ (%)/ N Controls ^c^ (%)	OR (95% CI)	*p*
*SLC22A1*	rs628031	Met408Leu	Recessive	G/G–A/G A/A	30 (24)/94 (76) 7 (58)/5 (42)	4.52 (1.33–15.43)	0.015
*ABCG1*	rs492338	Intron	Log-additive	0: T/T 1: C/T 2: C/C	16 (39)/25 (61) 16 (27)/43 (73) 5 (15)/28 (85)	0.54 (0.31–0.92)	0.021
*ABCB11*	rs496550	Intron	Log-additive	0: G/G 1: A/G 2: A/A	15 (38)/24 (62) 19 (26)/55 (74) 3 (13)/20 (87)	0.51 (0.28–0.94)	0.027
*ABCB11*	rs497692	Ala1028Ala	Log-additive	0: G/G 1: A/G 2: A/A	15 (38)/25 (62) 18 (25)/53 (75) 3 (13)/21 (87)	0.51 (0.28–0.94)	0.027
*CYP2D6*	rs1135840 ^d^	Ser486Thr	Log-additive	0: C/C 1: C/G 2: G/G	12 (22)/43 (78) 12 (24)/39 (76) 13 (46)/15 (54)	1.70 (1.03–2.82)	0.037
*CYP11B1*	rs7003319	3′UTR	Dominant	C/C A/C–A/A	11 (19)/48 (81) 26 (34)/51 (66)	2.32 (1.02–5.25)	0.038
*MAT1A*	rs4934027	Intron	Dominant	C/C C/T–T/T	31 (36)/55(64) 6 (12)/43 (88)	0.25 (0.1–0.65)	0.002

^a^: Selected by Akaike information criterion (AIC); ^b^: imatinib non-responders (failure to achieve MMR); ^c^: imatinib responders; ^d^: this gSNV did not fulfill HWE but was included due to its potential implication in CML/leukemogenesis.

**Table 3:** gSNVs significantly associated (*p* < 0.05) with MMR achievement with imatinib first-line treatment in the cross-validation analysis. Groups A, B, C, D and E correspond to one of the five analyses performed for this test.

Group	Gene	RefSNP ID	Model ^a^	Genotype	N Cases ^b^ (%)/ N Controls ^c^ (%)	OR (95% CI)	*p*
A	*MAT1A*	rs4934027	Dominant	C/C C/T–T/T	26 (36)/46 (64) 3 (8)/34 (92)	0.16 (0.04–0.56)	0.001
B	*CYP11B1*	rs7003319	Dominant	C/C C/A–A/A	6 (13)/39 (87) 24 (36)/42 (64)	3.71 (1.37–10)	0.006
B	*MAT1A*	rs4934027	Dominant	C/C C/T–T/T	24 (36)/43 (64) 6 (14)/37 (86)	0.29 (0.11–0.79)	0.009
C	*ABCG1*	rs492338	Log-additive	0: T/T 1: C/T 2: C/C	13 (38)/21 (62) 12 (27)/33 (73) 4 (15)/23 (85)	0.54 (0.3–0.99)	0.039
C	*CYP2D6*	rs1135840	Log-additive	0: C/C 1: C/G 2: G/G	8 (19)/33 (81) 9 (21)/33 (79) 12 (52)/11 (48)	2.11 (1.18–3.79)	0.010
C	*MAT1A*	rs4934027	Dominant	C/C C/T–T/T	26 (35)/48 (65) 3 (9)/30 (91)	0.18 (0.05–0.66)	0.003
C	*ABCB11*	rs495714	Log-additive	0: A/A 1: A/G 2: G/G	13 (43)/17 (57) 14 (24)/45 (76) 2 (10)/19 (90)	0.38 (0.19–0.77)	0.005
C	*SLC22A1*	rs628031	Log-additive	0: G/G 1: A/G 2: A/A	9 (21)/34 (79) 14 (25)/42 (75) 6 (67)/3 (33)	2.14 (1.05–4.35)	0.032
C	*ABCB11*	rs496550	Log-additive	0: G/G 1: G/A 2: A/A	13 (43)/17 (57) 15 (25)/46 (75) 1 (6)/16 (94)	0.35 (0.17–0.74)	0.004
C	*ABCB11*	rs497692	Log-additive	0:G/G 1: G/A 2: A/A	13 (42)/18 (58) 15 (25)/44 (75) 1 (6)/17 (94)	0.37 (0.18–0.76)	0.004
D	*ABCG1*	rs492338	Log-additive	0: T/T 1: C/T 2: C/C	13 (38)/21 (62) 14 (29)/35 (71) 3 (13)/20 (87)	0.53 (0.28–0.98)	0.038
D	*CYP2D6*	rs1135840	Log-additive	0: C/C 1: C/G 2: G/G	10 (21)/37 (79) 9 (23)/30 (77) 11 (50)/11 (50)	1.86 (1.07–3.25)	0.026
D	*MAT1A*	rs4934027	Dominant	C/C C/T–T/T	24 (37)/40 (63) 6 (14)/37 (86)	0.27 (0.1–0.73)	0.006
D	*ABCB11*	rs496550	Log-additive	0: G/G 1: A/G 2: A/A	12 (38)/20 (62) 16 (28)/41 (72) 2 (11)/17 (89)	0.51 (0.26–0.99)	0.04
D	*ABCB11*	rs497692	Log-additive	0: G/G 1: A/G 2: A/A	12 (36)/21 (64) 15 (28)/39 (72) 2 (10)/18 (90)	0.51 (0.26–0.99)	0.04
E	*ABCG1*	rs492338	Log-additive	0: T/T 1: C/T 2: C/C	14 (48)/15 (52) 13 (27)/36 (73) 3 (11)/24 (89)	0.37 (0.19–0.71)	0.002
E	*MAT1A*	rs4934027	Dominant	C/C C/T–T/T	24 (36)/43 (64) 6 (15)/34 (85)	0.32 (0.12–0.86)	0.017
E	*SLC22A1*	rs628031	Log-additive	0: G/G 1: A/G 2: A/A	8 (18)/37 (82) 16 (30)/37 (70) 6 (60)/4 (40)	2.44 (1.22–4.88)	0.009

^a^: Selected by Akaike information criterion (AIC); ^b^: imatinib non-responders (failure to achieve MMR); ^c^: imatinib responders.

**Table 4:** gSNVs significantly associated (*p* < 0.05) with MMR achievement in the permutation test (performed with a total of 10,000 iterations).

Gene	RefSNP ID	Model ^a^	*p*
*ABCG1*	rs492338	Log-additive	0.023
*CYP2D6*	rs1135840	Log-additive Recessive	0.032 0.016
*UGT2A1*	rs4148304	Recessive	0.012
*CYP11B1*	rs7003319	Dominant	0.047
*MAT1A*	rs4934027	Log-additive Dominant	0.003 0.002
*CYP2D6*	rs1065852	Recessive	0.003
*SLC22A1*	rs628031	Recessive	0.017
*ABCB11*	rs496550	Log-additive	0.027
*ABCB11*	rs497692	Log-additive	0.027

^a^: Selected by Akaike information criterion (AIC).

## Data Availability

The data present in this study can be partially available upon request to corresponding author in accordance with national regulations for data sharing.

## Supplementary Materials

The following supporting information can be downloaded at: https://www.mdpi.com/article/10.3390/jcm11206217/s1, Table S1: gSNVs significantly associated (*p* < 0.05) with MMR achievement to imatinib first-line treatment in the exploratory cohort (*n* = 45) and included in the analysis with the extended cohort; Table S2: cumulative incidences (median (95% CI)) of MMR achievement for each of the genotypes of the gSNVs statistically significant obtained in the analyses of the extended cohort (*n* = 137). *p*-value is indicated for each gSNV and those statistically significant are highlighted in bold.
