# Supplementary material for: Association between Germline Single-Nucleotide Variants in ADME Genes and Major Molecular Response to Imatinib in Chronic Myeloid Leukemia Patients
Source: J Clin Med. 2022 Oct 21;11(20):6217. doi: 10.3390/jcm11206217 (PMC9604607; doi:10.3390/jcm11206217)
Supplement: Supplementary file 1 [file jcm-11-06217-s001.zip › jcm-1935815-supplementary.pdf]

SUPPLEMENTARY MATERIAL

Table S1. gSNVs significantly associated (p<0.05) with MMR achievement to imatinib first-line treatment in the exploratory cohort (*n*=45) and included in the analysis with the extended cohort.

RefSNP ID	Gene	Gene name	ADME classification	Consequence
rs1134095	*CYP11B1*	Cytochrome P450 family 11 subfamily B member 1	Phase I enzyme	3'UTR variant
rs11770903	*PON3*	Paraoxonase 3	Other	Upstream variant
rs13959	*ALDH1A1*	Aldehyde dehydrogenase 1 family member A1	Phase II enzyme	Synonymous variant
rs2501870	*NR1I3*	Nuclear receptor subfamily 1 group I member 3	Other	Synonymous variant
rs3787537	*SLCO4A1*	Solute carrier organic anion transporter family member 4A1	Transporter	Intronic variant
rs492338	*ABCG1*	ATP binding cassette subfamily G member 1	Transporter	Intronic variant
rs6783962	*CHST13*	Carbohydrate sulfotransferase 13	Phase II enzyme	Intronic variant
rs7662029	*UGT2B7*	UDP glucuronosyltransferase family 2 member B7	Phase II enzyme	Upstream variant
rs1062033	*CYP19A1*	Cytochrome P450 family 19 subfamily A member 1	Phase I enzyme	Intronic variant
rs10509681	*CYP2C8*	Cytochrome P450 family 2 subfamily C member 8	Phase I enzyme	Missense variant
rs1135840	*CYP2D6*	Cytochrome P450 family 2 subfamily D member 6	Phase I enzyme	Missense variant
rs11807	*GSTM5*	Glutathione S-transferase mu 5	Phase II enzyme	3'UTR variant
rs1402467	*SULT1C4*	Sulfotransferase family 1C member 4	Phase II enzyme	Missense variant
rs2762934	*CYP24A1*	Cytochrome P450 family 24 subfamily A member 1	Phase I enzyme	3'UTR variant
rs4148304	*UGT2A1*	UDP glucuronosyltransferase family 2 member A2	Phase II enzyme	Missense variant
rs7003319	*CYP11B1*	Cytochrome P450 family 11 subfamily B member 1	Phase I enzyme	3'UTR variant
rs4925	*GSTO1*	Glutathione S-transferase omega 1	Phase II enzyme	Missense variant
rs7668258	*UGT2B7*	UDP glucuronosyltransferase family 2 member B7	Phase II enzyme	Upstream variant
rs2274405	*ABCC4*	ATP binding cassette subfamily C member 4	Transporter	Synonymous variant
rs1138541	*SLCO5A1*	Solute carrier organic transporter family member 5A1	Transporter	3'UTR variant
rs1202283	*ABCB4*	ATP binding cassette subfamily B member 4	Transporter	Synonymous variant
rs1530031	*CHST10*	Carbohydrate sulfotransferase 10	Phase II enzyme	3'UTR variant
rs2274406	*ABCC4*	ATP binding cassette subfamily C member 4	Transporter	Missense variant
rs28360521	*CYP2D6*	Cytochrome P450 family 2 subfamily D member 6	Phase I enzyme	Intronic variant
rs4543	*CYP11B2*	Cytochrome P450 family 11 subfamily B member 2	Phase I enzyme	Synonymous variant
rs4934027	*MAT1A*	Methionine adenosyltransferase 1A	Other	Intronic variant
rs700519	*CYP19A1*	Cytochrome P450 family 19 subfamily A member 1	Phase I enzyme	Missense variant
rs8050894	*VKORC1*	Vitamin K epoxide reductase complex subunit 1	Other	Intronic variant
rs1065852	*CYP2D6*	Cytochrome P450 family 2 subfamily D member 6	Phase I enzyme	Missense variant
rs11568591	*ABCC3*	ATP binding cassette subfamily C member 3	Transporter	Missense variant
rs1229984	*ADH1B*	Alcohol dehydrogenase 1B (class I), beta polypeptide	Phase II enzyme	Missense variant
rs1604741	*SULT1B1*	Sulfotransferase family 1B member 1	Phase II enzyme	Intronic variant
rs2276299	*SLC22A8*	Solute carrier family 22 member 8	Transporter	Synonymous variant
rs2884737	*VKORC1*	Vitamin K epoxide reductase complex subunit 1	Other	Intronic variant
rs495714	*ABCB11*	ATP binding cassette subfamily B member 11	Transporter	Intronic variant
rs2242046	*SLC28A1*	Solute carrier family 28 member 1	Transporter	Missense variant

rs1395	*SLC5A6*	Solute carrier family 5 member 6	Transporter	Missense variant
rs743616	*ARSA*	Arylsulfatase A	Other	Intronic variant
rs11764079	*PON3*	Paraoxonase 3	Other	Upstream variant
rs742350	*FMO1*	Flavin containing dimethylaniline monooxygenase 1	Phase II enzyme	Synonymous variant
rs8192879	*CYP7A1*	Cytochrome P450 family 7 subfamily A member 1	Phase I enzyme	3'UTR variant
rs3100	*UGT2B15*	UDP glucuronosyltransferase family 2 member B15	Phase II enzyme	3'UTR variant
rs2292334	*SLC22A3*	Solute carrier family 22 member 3	Transporter	Synonymous variant
rs17685	*POR*	Cytochrome p450 oxidoreductase	Other	3'UTR variant
rs12960	*SPG7*	SPG7 matrix AAA peptidase subunit, paraplegin	Other	Missense variant
rs11572080	*CYP2C8*	Cytochrome P450 family 2 subfamily C member 8	Phase I enzyme	Missense variant
rs1080983	*CYP2D6*	Cytochrome P450 family 2 subfamily D member 6	Phase I enzyme	Upstream variant
rs628031	*SLC22A1*	Solute carrier family 22 member 1	Transporter	Missense variant
rs496550	*ABCB11*	ATP binding cassette subfamily B member 11	Transporter	3'UTR variant
rs895729	*CHST1*	Carbohydrate sulfotransferase 1	Phase II enzyme	Intronic variant
rs4646	*CYP19A1*	Cytochrome P450 family 19 subfamily A member 1	Phase I enzyme	3'UTR variant
rs316019	*SLC22A2*	Solute carrier family 22 member 2	Transporter	Missense variant
rs2297322	*SLC15A1*	Solute carrier family 15 member 1	Transporter	Missense variant
rs1801246	*ATP7B*	ATPase copper transporting beta	Transporter	Synonymous variant
rs1296954	*GSTM5*	Glutathione S-transferase mu 5	Phase II enzyme	5'UTR variant
rs11584174	*NR1I3*	Nuclear receptor subfamily 1 group I member 3	Other	Synonymous variant
rs1080985	*CYP2D6*	Cytochrome P450 family 2 subfamily D member 6	Phase I enzyme	Upstream variant
rs13226149	*PON3*	Paraoxonase 3	Other	Synonymous variant
rs9934438	*VKORC1*	Vitamin K epoxide reductase complex subunit 1	Other	Intronic variant
rs7438284	*UGT2B7*	UDP glucuronosyltransferase family 2 member B7	Phase II enzyme	Synonymous variant
rs497692	*ABCB11*	ATP binding cassette subfamily B member 11	Transporter	Synonymous variant
rs4679028	*SLC22A14*	Solute carrier family 22 member 14	Transporter	Intronic variant
rs324420	*FAAH*	Fatty acid amide hydrolase	Other	Missense variant
rs2359612	*VKORC1*	Vitamin K epoxide reductase complex subunit 1	Other	Intronic variant
rs212090	*ABCC1*	ATP binding cassette subfamily C member 1	Transporter	3'UTR variant
rs11731028	*SULT1B1*	Sulfotransferase family 1B member 1	Phase II enzyme	Intronic variant
rs1109407	*SLCO2B1*	Solute carrier organic transporter family member 2B1	Transporter	Synonymous variant
rs2242416	*SLC22A7*	Solute carrier family 22 member 7	Transporter	Downstream variant
rs7439366	*UGT2B7*	UDP glucuronosyltransferase family 2 member B7	Phase II enzyme	Missense variant
rs5303	*CYP11B1*	Cytochrome P450 family 11 subfamily B member 1	Phase I enzyme	3'UTR variant
rs4683739	*CHST2*	Carbohydrate sulfotransferase 2	Phase II enzyme	Upstream variant
rs3775770	*SULT1E1*	Sulfotransferase family 1E member 1	Phase II enzyme	Intronic variant
rs2479390	*GSTM5*	Glutathione S-transferase mu 5	Phase II enzyme	Intronic variant
rs1126692	*FMO1*	Flavin containing dimethylaniline monooxygenase 1	Phase II enzyme	Synonymous variant
rs750398	*CHST1*	Carbohydrate sulfotransferase 1	Phase II enzyme	Synonymous variant
rs4736312	*CYP11B1*	Cytochrome P450 family 11 subfamily B member 1	Phase I enzyme	3'UTR variant

Table S2. Cumulative incidences (median [95%CI]) of MMR achievement for each of the genotypes of the gSNVs statistically significant obtained in the analyses of the extended cohort (*n*=137). *P*-value is indicated for each gSNV and those statistically significant are in bold.

Gene	RefSNP ID	Genotype	Cumulative incidence (median [95%CI])	*p*
ABCG1	rs492338	T/T<br>C/T<br>C/C	58% [42% - 71%]<br>73% [62% - 81%]<br>83% [65% - 93%]	**0.046**
ABCB11	rs496550	G/G<br>A/G<br>A/A	62% [47% - 74%]<br>73% [61% - 81%]<br>86% [73% - 94%]	0.338
ABCB11	rs497692	G/G<br>A/G<br>A/A	62% [47% - 74%]<br>73% [61% - 81%]<br>86% [86% - 86%]	0.362
ABCB11	rs495714	G/G<br>A/G<br>A/A	81% [67% - 89%]<br>74% [63% - 83%]<br>62% [47% - 74%]	0.344
CYP2D6	rs1135840	C/C<br>C/G<br>G/G	77% [65% - 85%]<br>75% [61% - 85%]<br>52% [34% - 67%]	0.148
CYP11B1	rs7003319	C/C<br>A/C<br>A/A	81% [69% - 88%]<br>66% [54% - 76%]<br>60% [42% - 74%]	0.179
CYP11B1	rs7003319<br>(dominant)	C/C<br>A/C- A/A	81% [69% - 88%]<br>64% [54% - 73%]	0.069
MAT1A	rs4934027	C/C<br>C/T<br>T/T	62% [52% - 70%]<br>86% [72% - 93%]<br>100% [100% - 100%]	**0.011**
MAT1A	rs4934027<br>(dominant)	C/C<br>C/T-T/T	62% [52% - 70%]<br>88% [75% - 94%]	**0.011**
SLC22A1	rs628031	G/G<br>A/G<br>A/A	76% [64% - 85%]<br>73% [61% - 82%]<br>36% [11% - 63%]	0.119
SLC22A1	rs628031<br>(recessive)	G/G-A/G<br>A/A	75% [67% - 81%]<br>36% [11% - 63%]	**0.043**
CYP2D6	rs1065852	C/C<br>C/T<br>T/T	69% [59% - 77%]<br>68% [52% - 80%]<br>100% [100% - 100%]	**0.047**
CYP2D6	rs1065852<br>(recessive)	C/C-C/T<br>T/T	69% [60% - 76%]<br>100% [100% - 100%]	**0.014**
UGT2A1	rs4148304	G/G<br>A/G<br>A/A	72% [62% - 79%]<br>80% [69% - 88%]<br>20% [20% - 20%]	0.209
UGT2A1	rs4148304<br>(recessive)	G/G-A/G<br>A/A	74% [66% - 80%]<br>20% [20% - 20%]	0.124
